# Neighborhood social cohesion and psychological distress among caregiving spouses with or without a nearby social support network

**DOI:** 10.3389/fpubh.2026.1774526

**Published:** 2026-04-28

**Authors:** Yeon Jin Choi, Seungjong Cho, Jihyeong Jeong, Lirisha Tuladhar

**Affiliations:** 1 College of Social Work, University of Kentucky, Lexington, KY, United States; 2 Department of Sociology, Anthropology, and Social Work, Texas Tech University, Lubbock, TX, United States; 3 School of Social Work, University of Maryland, Baltimore, MD, United States

**Keywords:** aging, anxiety, caregiving, depressive symptoms, hopelessness, mental health, neighborhood characteristics, social environments

## Abstract

**Background:**

Caregiving spouses often experience substantial physical and emotional strain, placing them at elevated risk for psychological distress. While nearby social support networks and neighborhood social cohesion have each been linked to caregiver well-being, neighborhood social cohesion may serve as a complementary resource, particularly for those without nearby social support. However, little is known about whether the association between neighborhood social cohesion and psychological distress varies depending on the availability of nearby social support among caregiving spouses.

**Methods:**

Using data from the 2006–2012 waves of the Health and Retirement Study, this study examined associations between perceived neighborhood social cohesion and psychological distress among caregiving spouses of older adults (*N* = 1,375), stratified by nearby social support network status. Regression models were estimated to assess associations of neighborhood social cohesion with anxiety, hopelessness, and depressive symptoms, adjusting for sociodemographic characteristics and caregiving intensity.

**Results:**

Approximately 13.0% of caregiving spouses reported having no nearby social support network. Caregivers without nearby social support reported higher levels of anxiety, hopelessness, and depressive symptoms compared with those who had nearby social support networks. Across all outcomes, higher neighborhood social cohesion was significantly associated with lower psychological distress in both groups. However, associations were consistently stronger among caregivers lacking nearby social support networks (anxiety: *b* = −0.12, *p* < 0.001; hopelessness: *b* = −0.42, p < 0.001; depressive symptoms: *b* = −0.26, *p* < 0.05) than among those with nearby social support networks (anxiety: *b* = −0.05, *p* < 0.01; hopelessness: *b* = −0.16, *p* < 0.001; depressive symptoms: *b* = −0.12, *p* < 0.01).

**Conclusion:**

These findings highlight the importance of neighborhood-level social environments as a potential resource for caregiver mental health, particularly for caregivers with limited proximate social support. They further suggest that community-based efforts to strengthen social cohesion may complement caregiver support strategies and enhance psychological well-being among caregiving spouses.

## 1 Introduction

In the United States, a growing proportion of older adults live with disabilities or chronic conditions (1–3), a trend driven by longer life expectancy and an extended period of living with disability (4, 5). In 2023, 93% of older adults aged 65 and over reported having at least one chronic condition (2). As the number of older adults living with disabilities or chronic conditions continues to grow, so too does the need for caregiving.

Family members have long been the primary source of support for older adults with disabilities or chronic conditions. In 2022, an estimated 24.1 million family caregivers provided care to older adults in the United States (6). Among them, spouses often take on the most intensive and prolonged caregiving responsibilities (7). Prior research consistently shows that caregiving spouses devote more hours to providing high-intensity care (8–10). Because of this, they are more likely to experience social isolation and loneliness, heightened emotional strain, stress, and psychological distress (9, 11, 12), as well as elevated risk of physical and mental health problems (13–15). Identifying factors that may protect caregiving spouses from psychological distress is therefore critical.

The stress process model (16) provides a widely used framework for understanding how caregiving stressors influence health outcomes. According to this model, caregiver stress emerges from a dynamic interplay of stressors, resources, and contextual conditions that vary across individuals and shape their psychological well-being. While caregiving represents a chronic and potentially persistent stressor, access to psychosocial and environmental resources may mitigate stress exposure and attenuate adverse mental health outcomes (16). Within this framework, social support and contextual resources are central mechanisms through which the psychological consequences of caregiving stress may be buffered.

An individual’s social support network constitutes a key contextual resource in this framework and plays an important role in caregiving spouses’ psychological well-being, as it provides emotional, instrumental, and informational assistance. In particular, the residential proximity of children, relatives, and friends fosters opportunities for personal involvement and interaction (17, 18) and facilitates help with daily tasks and caregiving responsibilities (19, 20), which may reduce caregiving burden and stress (21, 22). Empirical evidence consistently underscores the protective role of social support in caregiver mental health. In their systematic and meta-analysis, Gutiérrez-Sánchez et al. (23) demonstrated that social support plays a clinically meaningful role in caregiver well-being and serves as a key protective factor against depressive symptoms among informal caregivers. Focusing specifically on older caregivers, Muñoz-Bermejo et al. (24) found that higher levels of perceived social support were associated with more favorable mental health outcomes and reduced stress. Similarly, Chen et al. (25) reported that lower levels of social capital, characterized by the absence of social and familial relationships within the residential community, were associated with higher levels of depression among older caregivers of people living with dementia.

However, not all caregivers have access to geographically proximate social ties. For those lacking nearby social support networks, neighborhood social cohesion may serve as a complementary resource. Neighborhood social cohesion refers to the sense of trust, connection, and mutual support among residents within their local community (26). Cohesive neighborhoods foster emotional closeness and provide opportunities for instrumental assistance, such as transportation or help with household tasks, thereby strengthening social bonds and community attachment (27). In addition, strong neighborhood social cohesion promotes more frequent face-to-face interactions among residents (28), creating opportunities for caregivers to exchange information and engage in reciprocal, community-level support (29). Supporting this perspective, Kim et al. (30) reported that greater neighborhood social capital and cohesion were associated with fewer challenges in accessing services for informal caregivers of older adults. Empirical evidence further confirms the protective benefits of neighborhood social cohesion for caregiving spouses (31, 32). For example, Choi and Ailshire (32) found that higher perceived cohesion was associated with fewer depressive symptoms among caregiving spouses, attributing this relationship to increased community engagement, social interaction, and the exchange of emotional and instrumental support. Similarly, Lee and Marier (31) reported that greater neighborhood cohesion was linked to reduced depressive symptoms and improved cognitive functioning. Collectively, these findings suggest that neighborhood social cohesion may benefit caregivers broadly and may be particularly important for those without geographically proximate social support networks, serving as an alternative source of support that helps safeguard mental health.

Despite these potential benefits, neighborhood social cohesion has received limited empirical attention among caregiving spouses. Moreover, no prior studies have examined whether the association between neighborhood social cohesion and psychological distress differs depending on the availability of nearby social support networks. Clarifying this relationship is critical for identifying protective factors that promote mental well-being in this population. Accordingly, this study examines the relationship between neighborhood social cohesion and psychological distress among caregiving spouses, stratified by the presence or absence of a nearby social support network.

## 2 Materials and methods

### 2.1 Data and sample

This study used data from the Health and Retirement Study (HRS)^1^, a publicly available longitudinal panel study of approximately 20,000 nationally representative Americans over the age of 50 and their spouses. The HRS has collected extensive information on respondents’ sociodemographic characteristics, social relationships, health status, and well-being biennially since 1992.

For the present study, data from the 2006–2012 waves were analyzed, as these waves included the most comprehensive assessments of psychological distress and residents’ evaluations of neighborhood social environments. Information on respondents’ psychological well-being and neighborhood environments was obtained via the self-administered Psychosocial and Lifestyle Questionnaire (PLQ; also referred to as the Leave-Behind Questionnaire). The PLQ was first administered in 2006 to a random half of the sample, with the remaining half completing it in 2008, and this alternating design continued in subsequent waves (33). To ensure temporal consistency, all variables included in the analysis were drawn from the same wave in which the respondent completed the PLQ.

Our sample consisted of caregiving spouses. A respondent was defined as a caregiving spouse if their spouse had at least one limitation in activities of daily living (ADL) or instrumental activities of daily living (IADL) and identified the respondent as their primary caregiver, consistent with previous studies (32, 34). The 2006–2012 HRS includes 1,726 caregiving spouses who completed the PLQ. From this group, we excluded respondents who were not living with their spouse (*n* = 45) or who are residing in nursing homes (*n* = 22). After further excluding respondents with missing data on key variables and covariates (*n* = 284), the final analytic sample included 1,375 participants. Respondents with missing data were excluded using listwise deletion. Survey-weighted comparisons indicated no significant difference between included and excluded respondents in psychological distress, sociodemographic characteristics, or caregiving intensity, with the exception that excluded respondents were slightly older.

### 2.2 Measures

#### 2.2.1 Psychological distress

To capture multiple aspects of psychological distress experienced by caregiving spouses, we included three measures: anxiety, hopelessness, and depressive symptoms.

##### 2.2.1.1 Anxiety

Anxiety was measured using five items adapted from the Beck Anxiety Inventory (BAI). Respondents were asked how often they experienced specific symptoms of anxiety during the past week, including: “I had fear of the worst happening,” “I was nervous,” “I felt my hands trembling,” “I had a fear of dying,” and “I felt faint.” Each item was rated on a four-point scale (1 = Never to 4 = Most of the time). Scores across the five items were averaged to create an overall anxiety index, with higher scores reflecting greater anxiety symptoms. Cases with more than two missing responses were coded as missing.

##### 2.2.1.2 Hopelessness

Hopelessness was measured using four items. The items were drawn from Everson et al. (35) and Beck et al. (36) and asked respondents to indicate how much they agreed or disagreed with the following statements: “I feel it is impossible for me to reach the goals that I would like to strive for,” “The future seems hopeless to me and I cannot believe that things are changing for the better,” “I do not expect to get what I really want,” and “There’s no use in really trying to get something I want because I probably will not get it.” Each item was rated on a 6-point Likert scale (1 = Strongly disagree to 6 = Strongly agree). A mean score was calculated across the four items to represent overall hopelessness, with higher scores reflecting greater feelings of hopelessness. Cases with more than two missing responses were coded as missing on the composite score.

##### 2.2.1.3 Depressive symptoms

Depressive symptoms were measured using the eight-item short form of the Center for Epidemiologic Studies Depression Scale (CES-D; range: 0–8) (37). The items assess both negative (e.g., feeling depressed, lonely, sad, or that everything is an effort) and positive affect (feeling happy and enjoying life). Respondents reported how often they experienced each feeling during the past week. Positive items were reverse-coded, and responses were summed, with higher scores indicating greater depressive symptoms.

#### 2.2.2 Neighborhood social cohesion

Perceived neighborhood social cohesion was measured using four items. Respondents were asked how they felt about their local area, defined as within about a mile or a 20-min walk from their home. Items included statements such as “I really feel part of this area,” “Most people in this area can be trusted,” “Most people in this area are friendly,” and “If you were in trouble, there are lots of people in this area who would help you.” Each item was rated on a seven-point scale (1 = Strongly disagree to 7 = Strongly agree). Responses were averaged to form a composite index of neighborhood social cohesion, with higher scores indicating stronger perceived social cohesion. Cases with more than two missing items were coded as missing. This measure has been widely used in prior studies and demonstrated strong construct validity in capturing collective social resources at the neighborhood level (26, 31, 32).

#### 2.2.3 Nearby social support network

The presence of a nearby social support network was determined based on whether respondents reported having children, relatives, or friends living in their neighborhood. Respondents who had at least one of these types of social ties were coded as “Yes,” whereas those without any nearby children, relatives, or friends were coded as “No.” This binary operationalization captures the presence versus absence of geographically proximate informal social ties that are most likely to provide timely emotional or instrumental assistance. This approach is consistent with prior research emphasizing the importance of geographic proximity in caregiving support [e.g., (17, 18, 20)].

#### 2.2.4 Covariates

Sociodemographic covariates included age, gender (male; female), race/ethnicity (non-Hispanic White; non-Hispanic Black; non-Hispanic others; Hispanic), educational attainment (less than high school; high school; more than high school), household asset (decimal), self-reported health of caregiver (range: 0 = poor to 5 = excellent), and residential urbanicity (urban; suburban; exurban). Based on previous studies reporting the influence of caregiving intensity on caregivers’ psychological well-being (7, 32, 34), caregiving intensity was also included. Caregiving intensity was assessed using two indicators: type of care provided (IADLs only; ADLs only; both IADLs and ADLs) and caregiving hours per day (range: 0–24).

### 2.3 Analysis plan

We first present the sample characteristics. We then estimated regression models to examine the association of neighborhood social cohesion with anxiety, hopelessness, and depressive symptoms, stratified by the presence or absence of a nearby social support network. Because some respondents have multiple observations (maximum = 2), we report cluster-adjusted standard errors. Sample weights provided by the HRS were applied in all analyses to account for the complex survey design and sample composition. Analyses were conducted using Stata 18.

## 3 Results

### 3.1 Sample characteristics

Table 1 presents the sample characteristics for the full sample and stratified by nearby social support network status. The mean anxiety score was 1.70 [Standard Deviation (SD) = 0.66] on a 4-point scale (range: 1–4), indicating relatively low levels of anxiety. The mean hopelessness score was 2.66 (SD = 1.34) on a 6-point scale (range: 1–6), suggesting moderate levels of hopelessness. The average number of depressive symptoms was 1.72 (SD = 2.10; range: 0–8). Most caregivers (87.0%) reported having a nearby social support network. The mean score of neighborhood social cohesion was 4.39 (SD = 1.41) on a 7-point scale (range: 1–7), indicating moderate to moderately high levels of cohesion. The mean age of the sample was 67.00 years (SD = 10.03). Slightly more than half of the sample were female (52.8%), the majority were non-Hispanic White (79.2%), 39.7% had more than high school education (39.7%), and 40.0% resided in urban areas. The mean household asset (decimal) was 5.33 (SD = 2.82). The mean self-reported health was 3.02 (SD = 1.08) on a 5-point scale (range: 1–5), indicating generally good health. Regarding caregiving characteristics, nearly half of the sample (49.7%) provided assistance with both IADLs and ADLs, with an average of 4.25 h of care per day (SD = 6.03).

**Table 1:** Sample characteristics (*N* = 1,375).

Variables	Whole sample	Without a nearby social support network	With nearby social support networks
	*M* (SD)/%	*M* (SD)/%	*M* (SD)/%
Anxiety (range: 1–4)	1.70 (0.66)	1.72 (0.63)	1.70 (0.66)
Hopelessness (range: 1–6)	2.66 (1.34)	2.86 (1.41)	2.63 (1.32)
Depressive symptoms (range: 0–8)	1.72 (2.10)	1.82 (2.23)	1.70 (2.07)
Nearby social support network			
No	13.0		
Yes	87.0		
Neighborhood social cohesion (range: 1–7 high)	4.39 (1.41)	3.80 (1.42)	4.47 (1.39)
Age	67.00 (10.03)	63.47 (9.18)	67.5 (10.06)
Gender			
Male	47.2	50.3	46.8
Female	52.8	49.7	53.2
Race/ethnicity			
Non-Hispanic white	79.2	75.1	80.0
Non-Hispanic black	7.9	10.9	7.4
Non-Hispanic others	3.7	5.5	3.4
Hispanic	9.4	8.5	9.5
Education			
Less than high school	18.8	10.6	20.0
High school	39.7	32.2	40.9
More than high school	41.5	57.2	39.1
Household asset (decimal)	5.33 (2.82)		
Self-reported health (range: 1 poor–5 excellent)	3.02 (1.08)	3.09 (0.97)	3.01 (1.10)
Residential urbanicity			
Urban	40.0	40.2	40.0
Suburban	25.1	25.3	25.1
Exurban	34.8	34.5	34.9
Type of care			
IADL only	30.2	28.3	30.5
ADL only	19.6	23.2	19.0
Both IADL and ADL	50.2	48.5	50.5
Hours of care (range: 1–24)	4.25 (6.04)	4.27 (6.00)	4.24 (6.05)

*M*, Mean; SD, standard deviation; IADL, instrumental activities of daily living; ADL, activities of daily living.

When stratified by nearby social network status, caregivers without a nearby social support network reported higher levels of anxiety [Mean (*M*) = 1.72, SD = 0.63], hopelessness (*M* = 2.86, SD = 1.41), and depressive symptoms (*M* = 1.82, SD = 2.23) compared with those who had nearby social support networks (anxiety: *M* = 1.70, SD = 0.66; hopelessness: *M* = 2.63, SD = 1.32; depressive symptoms: *M* = 1.70, SD = 2.07). They also reported lower neighborhood social cohesion (*M* = 3.80, SD = 1.42) than caregivers with nearby social support network (*M* = 4.47, SD = 1.39).

### 3.2 Nearby social networks, neighborhood social cohesion, and psychological distress

Tables 2–4 presents regression results predicting anxiety, hopelessness, and depressive symptoms, stratified by nearby social support network status. Across all outcomes, higher neighborhood social cohesion was significantly associated with lower levels of anxiety, hopelessness, and depressive symptoms among caregiving spouses, regardless of nearby social support network status. However, the magnitude of these associations were consistently stronger among caregiving spouses without a nearby social support network [anxiety: *b* = −0.12, Standard Error (SE) = 0.03, *p* < 0.001; hopelessness: *b* = −0.42, SE = 0.07, *p* < 0.001; depressive symptoms: *b* = −0.26, SE = 0.12, *p* < 0.05] compared with those who had a nearby social support network (anxiety: *b* = −0.05, SE = 0.02, *p* < 0.01; hopelessness: *b* = −0.16, SE = 0.03, *p* < 0.001; depressive symptoms: *b* = −0.12, SE = 0.05, *p* < 0.05).

**Table 2:** Regression models predicting anxiety among caregiving spouses by nearby social support network status (*N* = 1,375).

Variables	Without a nearby social support network		With nearby social support networks	
	Coef.	Std. err.	Coef.	Std. err.
Neighborhood social cohesion (range: 1–7 high)	−0.12^***^	(0.03)	−0.05^**^	(0.02)
Age	0.00	(0.01)	−0.01^*^	(0.00)
Gender (ref: male)				
Female	0.17	(0.11)	0.11^*^	(0.04)
Race/ethnicity (ref: non-Hispanic white)				
Non-Hispanic black	−0.34^+^	(0.20)	0.03	(0.07)
Non-Hispanic others	−0.03	(0.28)	−0.13	(0.12)
Hispanic	−0.13	(0.17)	−0.09	(0.10)
Education (ref: less than high school)				
High school	−0.27	(0.20)	−0.04	(0.07)
More than high school	−0.09	(0.18)	−0.04	(0.07)
Household asset (decimal)	−0.00	(0.02)	−0.02^+^	(0.01)
Self-reported health (range: 1 poor–5 excellent)	−0.21^**^	(0.06)	−0.21^***^	(0.02)
Residential urbanicity (ref: urban)				
Suburban	−0.06	(0.11)	0.04	(0.05)
Exurban	−0.03	(0.12)	0.10^*^	(0.05)
Type of care (ref: IADL only)				
ADL only	0.30^*^	(0.13)	0.03	(0.06)
Both IADL and ADL	0.33^**^	(0.12)	0.04	(0.05)
Hours of care (range: 0–24)	−0.01	(0.01)	0.00	(0.00)
Constant	2.48^***^	(0.45)	2.79^***^	(0.20)

IADL, instrumental activities of daily living; ADL, activities of daily living. ^+^*p* < 0.10; ^*^p < 0.05; ^**^p < 0.01; ^***^p < 0.001.

**Table 3:** Regression models predicting hopelessness among caregiving spouses by nearby social support network status (*N* = 1,375).

Variables	Without a nearby social support network		With nearby social support networks	
	Coef.	Std. err.	Coef.	Std. err.
Neighborhood social cohesion (range: 1–7 high)	−0.42^***^	(0.07)	−0.16^***^	(0.03)
Age	0.00	(0.01)	0.00	(0.00)
Gender (ref: male)				
Female	0.05	(0.26)	−0.11	(0.08)
Race/Ethnicity (ref: non-Hispanic white)				
Non-Hispanic black	−1.23^***^	(0.35)	−0.77^***^	(0.13)
Non-Hispanic others	−0.49	(0.57)	0.01	(0.24)
Hispanic	−0.66^+^	(0.35)	−0.05	(0.18)
Education (ref: less than high school)				
High school	−0.24	(0.47)	−0.22^+^	(0.13)
More than high school	−0.33	(0.43)	−0.47^***^	(0.13)
Household asset (decimal)	−0.04	(0.04)	−0.05^**^	
Self-reported health (range: 1 poor–5 excellent)	−0.17	(0.12)	−0.25^***^	(0.04)
Residential urbanicity (ref: urban)				
Suburban	−0.47^+^	(0.25)	0.02	(0.10)
Exurban	0.16	(0.30)	0.06	(0.10)
Type of care (ref: IADL only)				
ADL only	0.52	(0.32)	0.02	(0.12)
Both IADL and ADL	0.55^*^	(0.26)	0.10	(0.09)
Hours of care (range: 0–24)	0.02	(0.02)	−0.00	(0.01)
Constant	5.17^***^	(1.14)	4.66^***^	(0.41)

IADL, instrumental activities of daily living; ADL, activities of daily living. ^+^*p* < 0.10; ^*^p < 0.05; ^**^p < 0.01; ^***^p < 0.001.

**Table 4:** Regression models predicting depressive symptoms among caregiving spouses by nearby social support network status (*N* = 1,375).

Variables	Without a nearby social support network		With nearby social support networks	
	Coef.	Std. err.	Coef.	Std. err.
Neighborhood social cohesion (range: 1–7 high)	−0.26^*^	(0.12)	−0.12^*^	(0.05)
Age	−0.05^**^	(0.02)	−0.02^***^	(0.01)
Gender (ref: male)				
Female	0.60	(0.42)	0.43^**^	(0.12)
Race/ethnicity (ref: non-Hispanic White)				
Non-Hispanic black	−0.92	(0.72)	−0.04	(0.23)
Non-Hispanic others	−0.45	(0.72)	0.29	(0.47)
Hispanic	−0.82	(0.62)	0.05	(0.29)
Education (ref: less than high school)				
High school	−1.00	(0.62)	−0.20	(0.19)
More than high school	−0.42	(0.61)	−0.13	(0.20)
Household asset (decimal)	−0.06	(0.07)	−0.03	(0.03)
Self-reported health (range: 1 poor–5 excellent)	−0.47^*^	(0.21)	−0.78^***^	(0.07)
Residential urbanicity (ref: urban)				
Suburban	−1.03^*^	(0.42)	0.02	(0.16)
Exurban	−0.75	(0.46)	−0.19	(0.14)
Type of care (ref: IADL only)				
ADL only	0.67	(0.48)	0.03	(0.17)
Both IADL and ADL	0.97^*^	(0.39)	0.23^+^	(0.13)
Hours of care (range: 0–24)	0.07^*^	(0.03)	0.01	(0.01)
Constant	7.45^***^	(1.45)	5.74^***^	(0.61)

IADL, instrumental activities of daily living; ADL, activities of daily living. ^+^*p* < 0.10; ^*^p < 0.05; ^**^p < 0.01; ^***^p < 0.001.

## 4 Discussion

This study examined the association between neighborhood social cohesion and psychological distress among caregiving spouses, with particular attention to differences by the presence of a nearby social support network. Using a nationally representative sample from the HRS, we found that higher neighborhood social cohesion was consistently associated with lower levels of anxiety, hopelessness, and depressive symptoms. Although these associations were observed among caregivers both with and without nearby social support networks, they were substantially stronger among caregiving spouses who lacked proximate support from children, relatives, or friends.

These findings contribute to the caregiving and social environment literature in several important ways. First, they provide an evidence that neighborhood social cohesion may represent a meaningful psychosocial resource for caregiving spouses, complementing prior research that has primarily emphasized individual- and family-level sources of support [e.g., (34, 38)]. While close family members and friends remain critical sources of emotional and instrumental assistance, our results underscore that broader neighborhood contexts are also linked to caregivers’ psychological well-being, consistent with prior research (27, 29, 31, 32).

The stratified analyses further suggest that neighborhood social cohesion may be particularly salient for caregiving spouses who lack nearby social support networks. This pattern is consistent with stress process theory, which posits that supportive social and environmental resources can buffer the adverse psychological consequences of chronic stress exposure (16, 39). Differences in the magnitude of the association between neighborhood social cohesion and psychological distress may reflect variation in access to alternative sources of support. For caregivers without proximate ties, cohesive neighborhoods may represent an important source of social connection and support, potentially thereby alleviating feelings of loneliness and psychological distress (40, 41). In contrast, caregiving spouses with nearby networks may rely less on neighborhood-level support because more immediate sources of support are available. However, this does not imply that neighborhood social cohesion in unimportant for this subgroup. As the significant association between neighborhood social cohesion and psychological distress suggests, caregiving spouses with nearby networks may still benefit from multiple layers of social resources. Taken together, these findings highlight the importance of neighborhood-level social environments as a potential lever for supporting caregiver mental health. Community-level strategies that foster trust, social connectedness, and mutual support, such as neighborhood associations, community centers, caregiving support groups, age-friendly community initiatives, and other place-based programs, may contribute to strengthening neighborhood social cohesion and supporting psychological well-being among caregiving spouses. These approaches may be particularly relevant for caregiving spouses with limited access to nearby social support, a group at heightened risk for psychological distress and reduced informal assistance. From a practical standpoint, caregiver screening and support efforts may benefit from considering neighborhood context, and initiatives and interventions that promote local engagement and integration into neighborhood networks may represent scalable strategies for supporting older adults aging in place without proximate support.

### 4.1 Limitations

Despite these contributions, several limitations should be noted. First, although the HRS is longitudinal, the limited availability of repeated PLQ observations resulted in a cross-sectional design, which restricts causal inference. Although higher perceived neighborhood social cohesion was associated with lower psychological distress, it is possible that caregivers experiencing greater distress perceived their neighborhoods as less cohesive rather than the opposite. Second, reliance on self-reported measures may introduce reporting bias, as perceptions of neighborhood cohesion and psychological distress can be influenced by individual dispositions. Third, although analyses adjusted for sociodemographic characteristics and caregiving intensity, residual confounding cannot be ruled out. Lastly, the anxiety measure used in this study was only available in the HRS between 2006 and 2012, which required the use of data from the period. Because caregiving contexts may have changed over time, the findings should be interpreted within the context of the study period. Future research would benefit from longitudinal designs, the inclusion of objective measures of neighborhood environments, and examination of potential mechanisms through which neighborhood social cohesion influences caregiver well-being (e.g., perceived safety, collective efficacy, access to informal caregiving support), and the use of more recent data.

## 5 Conclusion

By examining neighborhood social cohesion and nearby social support within a unified framework, this study highlights the importance of layered social environments in understanding psychological distress among caregiving spouses. The findings suggest that neighborhood social cohesion may provide an additional layer of social support for caregivers with nearby social support networks and may be particularly relevant for those who lack proximate social ties, pointing to the role of broader community contexts in shaping caregiver well-being. These results support the integration of community-level perspectives into efforts to assist caregiving spouses, especially those with limited social support.

## Data Availability

The data used for this study is from the Health and Retirement Study (HRS), which is sponsored by the National Institute on Aging (NIA U01AG009740) and conducted by the University of Michigan. The datasets analyzed for this study is publicly available and can be downloaded from the HRS website: https://hrs.isr.umich.edu/.

## Footnotes

1
https://hrs.isr.umich.edu/about
